# Pectoralis muscle features assessed by chest CT and their associations with clinical and functional outcomes in children and adolescents with cystic fibrosis: a cross-sectional study

**DOI:** 10.36416/1806-3756/e20260116

**Published:** 2026-11-09

**Authors:** Mariana Severo da Costa, Marta Amor-Barbosa, Mariana Sbruzzi, Fernanda Maria Vendrusculo, Denise Lautenschleger Fischer, Flávia Moreira Carvalho, Rubens Gabriel Feijó Andrade, Leonardo Araújo Pinto, Márcio Vinícius Fagundes Donadio

**Affiliations:** 1. Laboratório de Atividade Física em Pediatria, Centro Infant, Pontifícia Universidade Católica do Rio Grande do Sul - PUCRS - Porto Alegre (RS) Brasil.; 2. Departament de Fisioteràpia, Facultat de Medicina i Ciències de la Salut, Universitat Internacional de Catalunya - UIC - Barcelona, Espanya.; 3. Centro de Diagnóstico por Imagem - CDI - Hospital São Lucas, Escola de Medicina, Pontifícia Universidade Católica do Rio Grande do Sul - PUCRS - Porto Alegre (RS) Brasil.; 4. Departamento de Pediatria, Centro Infant, Pontifícia Universidade Católica do Rio Grande do Sul - PUCRS - Porto Alegre (RS) Brasil.

**Keywords:** Cystic fibrosis, Tomography, X-ray computed, Muscle strength

## Abstract

**Objective:**

To evaluate the cross-sectional area and thickness of the pectoralis major muscle using chest CT and to investigate their associations with nutritional, respiratory, and functional parameters in children and adolescents with cystic fibrosis (CF).

**Methods:**

This was a cross-sectional study including patients 6-18 years of age with a confirmed diagnosis of CF. Anthropometric data were collected, as were data related to lung function and muscle strength assessed by dynamometry, as well as pectoralis muscle area and thickness measured by chest CT. Aerobic fitness was assessed with cardiopulmonary exercise testing.

**Results:**

The mean age of the participants was 11.2 ± 3.3 years, and the mean BMI was 17.1 ± 2.8 kg/m^2^. Oxygen pulse was found to correlate significantly with pectoralis muscle area (r = 0.51; p = 0.02), FEV_1_ (r = −0.58; p = 0.01), and BMI z-score (r = 0.60; p < 0.01). Pectoralis muscle area showed a moderate correlation with muscle strength (r = 0.42; p = 0.03), whereas muscle thickness showed only a weak association with FEV_1_.

**Conclusions:**

Our findings suggest that the cross-sectional area of the pectoralis muscle is a more relevant indicator of muscle function than is thickness. Integrating muscle morphology, strength, and aerobic fitness may contribute to a more comprehensive clinical assessment in patients with cystic fibrosis.

## INTRODUCTION

Cystic fibrosis (CF) is a chronic, progressive, genetic disease caused by dysfunction of the cystic fibrosis transmembrane conductance regulator (CFTR) gene, which is responsible for chloride transport across the cell membrane.^1^ That dysfunction affects multiple organs and systems, particularly the respiratory and gastrointestinal tracts.^2^ The progressive, multisystem nature of the disease can lead to weight loss, which is associated with decreased peripheral muscle mass and muscle strength, resulting in limitations in physical exercise and impaired quality of life.^3^ ^,^ ^4^

Although loss of muscle mass and strength has been associated with several clinical factors that may lead to complications in patients with CF, including reduced lung function (as determined by measuring FEV_1_), the assessment of skeletal muscle function is not routinely included in clinical evaluations of patients with CF.^5^ One of the most commonly used methods to assess muscle strength is isometric dynamometry, which measures force magnitude and identifies potential imbalances between muscle groups.^6^ ^,^ ^7^ However, dynamometry requires full patient cooperation, which restricts its applicability in specific situations, including young children, critically ill patients, and patients with impaired consciousness.^8^

For the evaluation of patients with CF, a CT scan is considered a standard imaging examination and, together with magnetic resonance imaging, is regarded as the gold standard for quantitative assessments of body composition.^9^ However, the use of CT in the evaluation of skeletal muscle remains limited, because it is primarily applied to detect structural lung changes.^10^ ^,^ ^11^ Chest CT-derived parameters related to the pectoralis muscle (density, cross-sectional area, and the pectoralis muscle index) have been described as predictors of clinical outcomes such as length of hospital stay and mortality in several diseases, as well as indicators of muscle quality, reflecting the composition and functional capacity of the muscle.^12^ Therefore, chest CT may serve as a useful tool, not only for pulmonary assessment but also for improving the understanding of muscle-related changes.^13^

The inclusion of an additional measurement in an examination that is already routinely performed may reduce the burden of multiple assessments and improve understanding of the relationship between muscle characteristics and the clinical status of children and adolescents with CF. Therefore, this study used chest CT to analyze pectoralis muscle parameters and to examine their associations with BMI, respiratory variables, and functional parameters, including pectoralis muscle strength and aerobic fitness. We also explored correlations between aerobic fitness variables and muscle strength, lung function, and nutritional status in children and adolescents with CF.

## METHODS

This was a cross-sectional study conducted from 2022 through 2024. The study was approved by the local research ethics committee (Reference no. 5.621.543) and was carried out in accordance with the principles of the Declaration of Helsinki. The study sample consisted of patients diagnosed with CF who were regularly followed at the CF outpatient clinic of a tertiary care hospital that manages pediatric and adult patients. Children and adolescents between 6 and 18 years of age were included. Patients with osteoarticular or musculoskeletal conditions that could interfere with the assessment were excluded, as were those presenting with evidence of pulmonary exacerbation in the last seven days before testing and those who had previously undergone lung transplantation. To ensure imaging homogeneity and minimize potential batch effects related to equipment variability, we also excluded patients in whom the chest CT scan was acquired with a nonstandardized protocol or in a scanner different from that specified. None of the patients were receiving CFTR modulator therapy at the time of data collection. For the sample size calculation, we relied on a previous study conducted in healthy individuals that reported a moderate correlation between CT-derived pectoralis muscle cross-sectional area and skeletal muscle mass.^14^ To ensure a more conservative estimation, we powered the study to detect a minimum expected correlation coefficient of 0.55 between the pectoralis muscle area measured by chest CT and pectoralis muscle strength assessed by dynamometry. Assuming a two-sided significance level of 5% (α = 0.05) and a statistical power of 80% (β = 0.20), the sample size required was estimated at 24 participants.

After confirmation of eligibility and completion of the informed consent process, including the acquisition of written informed consent from parents or legal guardians and of assent from the participants, previously obtained chest CT scans were identified. Because chest CT is routinely performed in this population, images were retrieved from electronic medical records and were considered eligible when performed within 3 months of all other variable assessments. Subsequently, anthropometric measurements (weight, height, and skinfold thickness) were made, after which participants sequentially underwent spirometry, digital dynamometry, and cardiopulmonary exercise testing (CPET). A 30-min rest period was provided to allow adequate recovery between the muscle strength assessment and CPET. Except for the chest CT examinations, all evaluations were carried out on the same day.

The primary study variables were pectoralis muscle cross-sectional area and thickness measured by chest CT, as well as pectoralis muscle strength measured by dynamometry. Secondary variables included aerobic fitness evaluated by CPET, lung function assessed by spirometry, and nutritional status determined by using age-adjusted BMI and skinfold thickness measurements. 

### Procedures

For the anthropometric assessment, body weight was measured with participants in the orthostatic position on a previously calibrated digital scale (Glass 1 FW; G-Tech, Rio de Janeiro, Brazil) with a precision of 100 g. Height was measured with a portable stadiometer (AlturaExata; TBW, São Paulo, Brazil) with a precision of 1 mm. The BMI was calculated by dividing the total body mass in kilograms by the square of height in meters (kg/m^2^). Nutritional status was classified based on BMI-for-age percentile, according to the reference distribution established by the US Centers for Disease Control and Prevention.^15^ The BMI z-score was calculated by using Epi Info software, version 3.5 (available at: http://wwwn.cdc.gov/epiinfo/).

Skinfold thickness was measured with a caliper (Innovare; Cescorf, Porto Alegre, Brazil), which exerts a pressure of 10 g/mm^2^ and has a precision of 1 mm. Measurements were taken on the right side of the body, at the triceps and subscapular sites. The triceps skinfold was measured at the midpoint between the acromion and olecranon on the posterior face of the arm, with the arm relaxed and hanging freely. The subscapular skinfold was measured approximately 2 cm below the inferior angle of the scapula, at an oblique angle, following the natural skinfold. Two measurements were obtained at each site, and the sum of the two measurements was used as an adiposity index, according to the criteria established by Jackson and Pollock.^16^

Lung function measurements were performed with a KoKo spirometer (Pulmonary Data Services, Inc., Louisville, CO, USA). The technical procedures and the criteria for acceptability and reproducibility of pulmonary function tests followed the recommendations of the American Thoracic Society/European Respiratory Society.^17^ Tests were performed with the participants in a seated position, wearing a nose clip and using a disposable mouthpiece. Each participant completed at least three acceptable, reproducible forced expiratory maneuvers, and the highest values of FVC and FEV_1_ were recorded. The spirometric parameters analyzed were FVC, FEV_1_, the FEV_1_/FVC ratio, and FEF_25-75%_. Spirometric data were normalized using the 2012 Global Lung Initiative reference equation.^18^

Pectoralis muscle strength was assessed with a handheld dynamometer (Med Force; MED DOR Ltda., Governador Valadares, Brazil) to measure maximal isometric pectoralis muscle strength.^19^ ^,^ ^20^ Patients performed shoulder adduction with the dominant limb, exerting maximal force against the dynamometer positioned on the medial region of the elbow. Each contraction was sustained for 5 s, and three trials were performed, with the highest value being considered the peak muscle strength. Muscle strength was initially expressed in kilograms and subsequently normalized to body surface area (BSA) to account for differences in body size. The BSA was estimated using the Mosteller formula, which has been validated across pediatric age ranges^21^:

BSA(m2)=√[(weight(kg)×height(cm))∕3600]

Muscle strength data were normalized by dividing the final measurement by the total BSA expressed in square meters, and are reported as kilogram-force per square meter.

Chest CT examinations were performed in a 64-slice scanner (Lightspeed VCT 64, General Electric, Milwaukee, WI, USA). Patients were placed in the supine position and instructed to perform a full inspiratory breath hold. No iodinated contrast agent was administered. Images were reconstructed by using a high-frequency spatial kernel with contiguous slices and a slice thickness of 1.00-1.25 mm. Quantitative assessment of the pectoralis muscle area was performed in a single axial slice immediately above the aortic arch, and muscle thickness was measured at the C4 vertebra level (Figure 1).^22^ The right and left pectoralis muscles were manually outlined, and their areas were calculated. For comparison with dynamometry, only measurements from the dominant side were included. Muscle area values were normalized to the BSA with the Mosteller formula, as previously described. Normalized values are expressed as square centimeters per square meter.^21^ ^,^ ^23^ Muscle thickness values were also normalized to the BSA by using the same formula and are expressed as centimeters per square meter.

**Figure 1:**
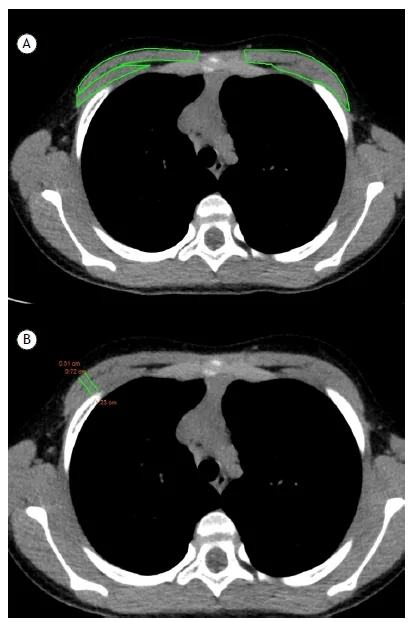
Axial chest CT images used for pectoralis muscle assessment. (A) Cross-sectional area measurement of the pectoralis muscle: the green contours represent the manual bilateral segmentation of the pectoralis major muscles. (B) Pectoralis muscle thickness measurement: the green marking indicates the measurement site, and the orange lines represent the linear thickness (in cm) measured perpendicular to the muscle plane at the predefined anatomical level.

The CPET involved the use of a metabolic testing system with a gas analyzer (VO2000; Medical Graphics Corporation, St. Paul, MN, USA), measuring gas uptake and providing information on ventilatory behavior every 10 s. The CPET was conducted by a trained researcher and followed the recommendations of the American College of Sports Medicine. A ramp protocol was applied as described by Barbosa e Silva et al.^24^ Data were recorded and processed with Aerograph software, version 4.3 (Micromed, Brasília, Brazil) for subsequent analysis. The CPET variables analyzed included peak VO_2_ (VO_2_peak), expressed in absolute values (L • min^-1^) and relative to body mass (mL • kg^-1^ • min^-1^), peak V_E_ (V_E_peak, in L • min^-1^), ventilatory equivalents for oxygen and carbon dioxide (VE/VO_2_ ratio and V_E_/VCO_2_ ratio, respectively), oxygen pulse (in mLO_2_ • beat^−1^), respiratory exchange ratio at peak exercise, breathing reserve (in %), and maximum HR (in bpm). Throughout the test, SpO_2_ was continuously monitored with a pulse oximeter (Nellcor; Covidien, Seoul, South Korea). Variables were analyzed at three time points: at rest (baseline), at the first ventilatory threshold, and at peak exercise. The ventilatory threshold was identified as the point at which the V_E_/VO_2_ ratio reached its minimum value and subsequently increased without a simultaneous increase in the V_E_/VCO_2_ ratio. Subjective perception of dyspnea was assessed with the Modified Borg Scale. The breathing reserve was calculated as the difference between the maximal voluntary ventilation and the V_E_peak, expressed as a percentage of the maximal voluntary ventilation. The test was considered maximal when at least two of the following criteria were met: inability to maintain the required speed; respiratory exchange ratio > 1.05; maximum HR > 85% of the age-predicted maximum (208 − [0.7 × age]); and the presence of a VO_2_ plateau, defined as an increase < 150 mL • min^-1^ in VO_2_ despite an increase in workload.^25^

### Statistical analysis

The distribution of the data was assessed with the Kolmogorov-Smirnov test and was found to be normal for all variables. Therefore, continuous variables are expressed as mean and standard deviation, whereas categorical variables are expressed as absolute and relative frequencies. Pearson’s correlation coefficient was used in order to evaluate the correlations that peripheral muscle features derived from chest CT showed with the variables of interest. Correlation strength was classified according to the absolute value of the correlation coefficient (r) as weak (r < 0.3), moderate (0.3 ≤ r < 0.7), or strong (r ≥ 0.7). The chi-square test was also used in order to assess associations between categorical variables. All analyses were performed with the Predictive Analytics Software package, version 18.0 (SPSS Inc., Chicago, IL, USA). The significance level was set at 5% (p < 0.05).

## RESULTS

A total of 46 patients were assessed for eligibility. Of those, 5 declined to participate and 15 were excluded for various reasons: because they were under 6 years of age (n = 7); because no acceptable CT scan was available (n = 6); and because the CT scan was acquired with a different protocol (n = 2). No patients were excluded due to pulmonary exacerbation. Therefore, the final sample comprised 26 patients 6-18 years of age diagnosed with CF. Anthropometric data were available for all included participants, as were measurements of muscle thickness, skinfold thickness, pulmonary function, and muscle strength. Of the 26 patients evaluated, 18 completed the CPET; the 8 remaining patients were excluded because of refusal to undergo the CPET (n = 5) or failure to meet the maximal exercise criteria (n = 3). The patient selection process is presented in Figure 2.

**Figure 2:**
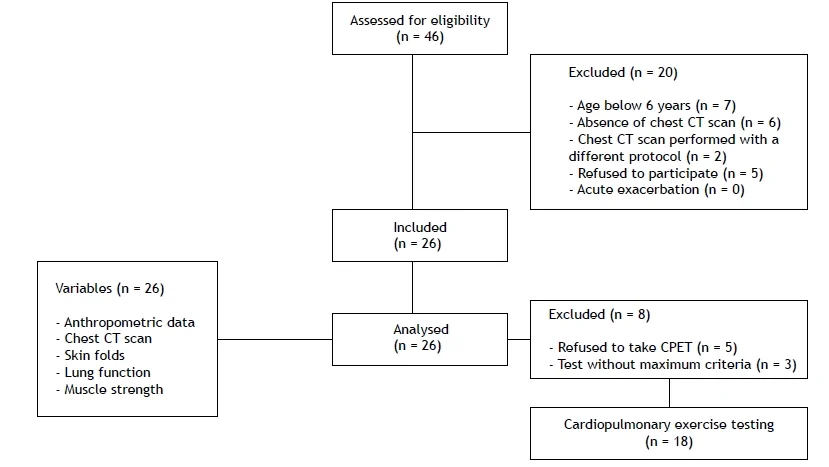
Flow chart of the patient selection process.

The mean age of the patients in the sample was 11.2 ± 3.27 years, and 17 (65.4%) of the patients were male. The ΔF508 homozygous mutation was present in 14 (53.8%) of the patients. Pancreatic insufficiency was observed in 25 patients (96.2%), and 5 (19.2%) had chronic *Pseudomonas aeruginosa* colonization. The mean BMI was 17.1 ± 2.8 kg/m^2^, the mean subscapular skinfold thickness was 13.1 ± 4.3 mm, and the mean triceps skinfold thickness was 20.8 ± 7.3 mm. The clinical and demographic characteristics of the participants are summarized in Table 1.

**Table 1:** Demographic, anthropometric, and clinical characteristics of the study sample.

Characteristic	(N = 26)
Age (years), mean ± SD	11.2 ± 3.3
Male, n (%)	17 (65.4)
Genotype, n (%)	
F508del homozygous	14 (53.8)
F508del heterozygous	10 (38.5)
Other	2 (7.7)
Height (cm), mean ± SD	144.6 ± 18.2
Weight (kg), mean ± SD	37.5 ± 15.1
BMI (kg/m^2^), mean ± SD	17.1 ± 2.8
BMI (z-score), mean ± SD	−0.41 ± 0.69
Subscapular skinfold (mm), mean ± SD	12.7 ± 4.1
Triceps skinfold (mm), mean ± SD	20.7 ± 6.6
Pancreatic insufficiency, n (%)	25 (96.2)
Chronic colonization with Pa, n (%)	5 (19.2)

Pa: *Pseudomonas aeruginosa*.

Lung function and muscle strength, as well as pectoralis muscle area and thickness measured by chest CT, are summarized in Table 2. The mean FEV_1_ z-score was −1.01 ± 1.49, and mean pectoralis muscle strength was 4.5 ± 1.07 kilogram-force/m^2^. The mean normalized pectoralis muscle area was 8.2 ± 2.9 cm^2^/m^2^, and the mean muscle thickness was 1.56 ± 0.42 cm/m^2^. The CPET variables are presented in Table 3. The mean VO_2_peak was 34.8 ± 4.4 mL • kg^-1^ • min^-1^. At the first ventilatory threshold, the mean VO_2_ corresponded to 62.6 ± 19.6% of the VO_2_peak. The mean V_E_peak was 44.4 ± 18.6 L • min^-1^.

**Table 2:** Characterization of lung function, muscle strength, and chest CT measurements.

Variable	(N = 26)
Lung function parameters, mean ± SD	
FEV_1_ (L)	1.99 ± 0.62
FEV_1_ (z-score)	−1.01 ± 1.49
FVC (L)	2.40 ± 0.84
FVC (z-score)	−0.64 ± 1.51
FEV_1_/FVC ratio	0.83 ± 0.07
FEF_25-75%_ (L • min^-1^)	2.21 ± 0.86
FEF_25-75%_ (z-score)	−0.75 ± 1.44
Isometric dynamometry findings, mean ± SD	
Pectoralis muscle strength (kgf)	5.50 ± 1.90
Pectoralis muscle strength (kgf/m^2^)	4.50 ± 1.07
Chest CT measurements, mean ± SD	
Total pectoralis muscle area (cm^2^)	10.40 ± 5.7
Normalized total pectoralis muscle area (cm^2^/m^2^)	8.20 ± 2.90
Pectoralis muscle thickness (cm)	1.88 ± 0.64
Normalized total pectoralis muscle thickness (cm/m^2^)	1.56 ± 0.42

kgf: kilogram-force.

**Table 3:** Cardiopulmonary exercise testing variables.

Variable	(N = 18)
At rest, mean ± SD	
HR (bpm)	90.7 ± 12.7
SpO_2_ (%)	97.8 ± 1.2
VO_2_ (L • min^-1^)	0.37 ± 0.12
VO_2_ (mL • kg^-1^ • min^-1^)	9.4 ± 3.2
At the first ventilatory threshold, mean ± SD	
HR (bpm)	140.3 ± 16.2
VO_2_ (L • min^-1^)	0.88 ± 0.36
VO_2_ (mL • kg^-1^ • min^-1^)	21.4 ± 6.2
VO_2_ (% of peak)	62.6 ± 19.6
V_E_ (L • min^-1^)	19.9 ± 9.1
V_E_/VO_2_	22.2 ± 2.6
V_E_/VCO_2_	24.4 ± 2.5
At peak exercise, mean ± SD	
HR (bpm)	186.5 ± 7.8
SpO_2_	96.8 ± 3.2
Respiratory exchange ratio	1,10 ± 0.07
VO_2_ (L • min^-1^)	1.44 ± 0.47
VO_2_ (mL • kg^-1^ • min^-1^)	34.8 ± 4.4
V_E_ (L • min^-1^)	44.4 ± 18.6
V_E_/VO_2_	30.1 ± 4.3
V_E_/VCO_2_	27.4 ± 3.4
O_2_ pulse (mLO_2_ • bpm^-1^)	7.8 ± 2.4
Breathing reserve (%)	46.8 ± 13.9

VT_1_: first ventilatory threshold.

The pectoralis muscle cross-sectional area showed a moderate, statistically significant positive correlation with pectoralis muscle strength (r = 0.42; p = 0.03). No significant associations were observed between the pectoralis muscle area and BMI z-score or FEV_1_. Muscle thickness showed a weak inverse correlation with FEV_1_ (r = −0.38; p = 0.05) but was not significantly associated with BMI or muscle strength. Correlation analyses are illustrated in Figure 3.

**Figure 3:**
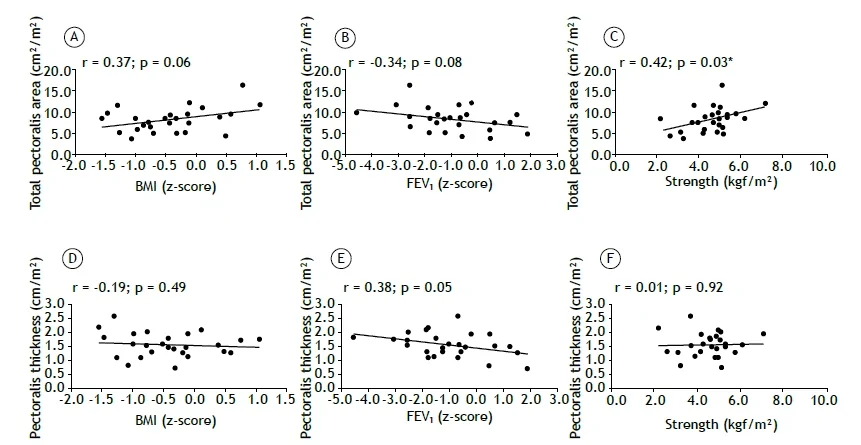
Correlations that clinical and functional variables showed with pectoralis muscle composition parameters. Correlations that CT-derived total pectoralis area showed with (A) BMI z-score, (B) FEV_1_ z-score, and (C) muscle strength. Correlations that CT-derived pectoralis thickness showed with (D) BMI z-score, (E) FEV_1_ z-score, and (F) muscle strength. kgf: kilogram-force. *Indicates statistical significance at p < 0.05.

Table 4 presents the CPET variables and their correlations with muscle strength, lung function, and nutritional status. Significant, moderate to strong correlations were identified: between V_E_ at the first ventilatory threshold and the BMI z-score (r = 0.56; p = 0.01); between the V_E_peak and the BMI z-score (r = 0.72; p = 0.001); between the peak V_E_/VO_2_ ratio and the BMI z-score (r = 0.52; p = 0.02); and correlations that the peak oxygen pulse showed with the muscle area (r = 0.51; p = 0.02), FEV_1_ (r = −0.58; p = 0.01) and with the BMI z-score (r = 0.60; p < 0.01).

**Table 4:** Correlations that cardiopulmonary exercise testing variables showed with indicators of body composition, lung function, and muscle strength.

Variable	Muscle area	Muscle thickness	Muscle strength	FEV_1_	BMI
				(z-score)	(z-score)
V_E_ VT_1_ (L.min^-1^)	r = 0.27; p = 0.27	r = −0.23; p = 0.34	r = 0.27; p = 0.27	r = 0.10; p = 0.68	r = 0.56; p = 0.01*
V_E_/VO_2_ VT_1_	r = −0.04; p = 0.86	r = −0.39; p = 0.10	r = 0.35; p = 0.14	r = 0.45; p = 0.06	r = −0.61; p = 0.81
V_E_/VCO_2_ VT_1_	r = 0.46; p = 0.85	r = −0.02; p = 0.91	r = 0.16; p = 0.50	r = 0.36 p = 0.13	r = −0.09; p = 0.71
VO_2_peak (% predicted)	r = −0.13; p = 0.59	r = −0.39; p = 0.10	r = −0.09; p = 0.70	r = 0.36; p = 0.13	r = 0.10; p = 0.67
HR max (bpm)	r = 0.96; p = 0.03	r = 0.31 p = 0.90	r = 0.04; p = 0.86	r = 0.26; p = 0.29	r = −0.08; p = 0.74
VO_2_ (mL.kg^-1^.min^-1^)	r = 0.15; p = 0.95	r = 0.26; p = 0.26	r = −0.10; p = 0.68	r = −0.15; p = 0.53	r = −0.06; p = 0.79
V_E_peak (L.min-^1^)	r = 0.50; p = 0.03*	r = 0.09; p = 0.70	r = 0.22; p = 0.37	r = −0.29; p = 0.24	r = 0.72; p < 0.01*
V_E_/VO_2_peak	r = 0.36; p = 0.14	r = 0.03; p = 0.88	r = 0.24; p = 0.33	r = 0.13; p = 0.59	r = 0.52*; p = 0.02
V_E_/VCO_2_peak	r = 0.24; p = 0.32	r = 0.07; p = 0.76	r = 0.19; p = 0.44	r = 0.26; p = 0.28	r = 0.21; p = 0.38
O_2_ pulse peak (mLO_2 ._bpm^-1^)	r = 0.51; p = 0.02*	r = 0.18; p = 0.47	r = 0.18; p = 0.47	r = −0.58; p = 0.01*	r = 0.60; p < 0.01*
Breathing reserve	r = −0.41; p = 0.08	r = −0.24; p = 0.32	r = −0.06; p = 0.81	r = 0.46; p = 0.52	r = −0.60; p < 0.01*

VT1: first ventilatory threshold; V_E_/VCO_2_: ventilatory equivalent for carbon dioxide production; and V_E_/VO_2_: ventilatory equivalent for oxygen uptake. *Indicates statistical significance at p < 0.05.

## DISCUSSION

In this study, we have evaluated the correlations that pectoralis muscle measurements assessed by chest CT (thickness and cross-sectional area) present with muscle strength, nutritional status, lung function, and aerobic fitness in patients with CF. Overall, increased pectoralis muscle area and thickness were associated with greater muscle strength and more favorable functional and respiratory responses during exercise, suggesting an integrated relationship among muscle mass, lung function, and aerobic fitness. Collectively, these findings suggest that thoracic muscle measurements derived from routine chest CT can reflect not only local muscle characteristics but also broader aspects of functional status and aerobic performance in this population.

Pectoralis muscle area showed a moderate, statistically significant correlation with muscle strength, indicating that greater muscle area was associated with greater strength. This finding is consistent with those of previous studies reporting moderate to strong correlations between muscle area and strength in patients with other musculoskeletal or oncologic conditions, such as lung cancer.^26^ ^,^ ^27^ However, the moderate strength of those correlations suggests that pectoralis muscle area alone is insufficient to serve as a surrogate marker of muscle strength in routine clinical practice. Further studies are needed in order to determine whether CT-derived muscle measurements can reliably inform clinical decision-making related to muscle function in patients with CF. In addition, several studies have demonstrated associations between pectoralis muscle area assessed by chest CT and clinically relevant outcomes, including those related to lung function and functional performance in other respiratory diseases, such as COVID-19, COPD, and pneumonia.^23^ ^,^ ^28^ ^,^ ^29^ In contrast, we did not find pectoralis muscle thickness to correlate significantly with muscle strength in our patient sample, a finding that may reflect its susceptibility to confounding factors such as subcutaneous fat, tissue thickness, and muscle architecture, all of which reduce its reliability as a functional indicator.^30^ Muscle cross-sectional area, in contrast, reflects a greater number of parallel muscle fibers and is therefore more directly related to functional capacity and physical performance,^31^ ^,^ ^32^ supporting its use as a more relevant functional indicator.

Although CT is primarily used for pulmonary assessment and is relatively costly, it also enables detailed evaluation of body composition, including the quantity and quality of skeletal muscle.^33^ Consequently, its use may be expanded to evaluations of nutritional status, risk of complications, and clinical prognosis,^33^ while also enabling longitudinal monitoring of muscle and bone mass, complementing traditional pulmonary evaluations. Prior studies have demonstrated the feasibility and clinical relevance of using chest CT for nutritional assessments, bone composition, and muscle mass evaluation, with implications for patient treatment and prognosis.^34^ These findings underscore the potential of this examination as a multifunctional tool that goes beyond traditional pulmonary analysis, allowing clinical assessment that is more comprehensive and individualized.^34^ ^-^ ^36^

An unexpected inverse correlation was observed between FEV_1_ and muscle thickness, suggesting that lower lung function may be associated with greater peripheral musculature. Although most evidence indicates a positive association between muscle mass and FEV_1_, particularly in athletes and healthy populations,^35^ this inverse pattern can occur in specific clinical contexts, such as isolated muscle conditioning programs in patients with COPD, where peripheral musculature improves ventilatory efficiency without altering FEV_1_.^36^ This finding supports the hypothesis that muscle area may be more strongly influenced by peripheral factors such as strength and physical conditioning than by lung function alone. Indeed, lung function parameters may not fully reflect physical performance or functional capacity, consistent with the findings of previous studies evaluating the dissociation between pulmonary function and exercise performance in chronic lung diseases.^37^

Among the correlations between pectoralis muscle measurements and CPET variables, two findings were particularly notable. The association between muscle area and V_E_peak suggested that individuals with greater muscle mass exhibit higher ventilatory capacity during maximal exercise. Increased functional muscle mass is associated with greater metabolic demand, requiring enhanced oxygen delivery and ventilation during exercise. In addition, greater muscle area was associated with a higher peak oxygen pulse. Oxygen pulse is an indirect marker of cardiovascular efficiency, and individuals with greater muscle mass may present higher oxygen uptake per heartbeat due to increased metabolic demand and better physical conditioning.^38^ In contrast, VO_2_peak did not correlate with pectoralis muscle measurements, which is physiologically plausible, given that VO_2_peak is a multifactorial variable influenced by multiple systems and primarily determined by larger muscles directly involved in exercise, such as the quadriceps.^39^

This study has several limitations. Although the target sample size was achieved, variability in the study population may have influenced the analyses, potentially limiting statistical power. That may be particularly relevant for the CPET outcomes, because the small final sample size suggests that these results should be interpreted with caution. In addition, the study was conducted at a single referral center, which could limit the generalizability of the findings. Nevertheless, the sample reflects the real-world clinical characteristics of pediatric patients with CF followed in outpatient settings. Furthermore, not all participants completed the CPET, which reduced the number of available observations. Finally, none of the participants were receiving CFTR modulator therapy at the time of data collection. Although that reflects the clinical context during the study period, future studies should investigate the impact of such therapy on muscle function and exercise capacity.

In conclusion, chest CT-derived assessment of the pectoralis muscle demonstrated a moderate correlation between pectoralis muscle area and muscle strength, whereas pectoralis muscle thickness showed no significant correlation with strength in children and adolescents with CF. Therefore, the cross-sectional area of the pectoralis muscle appears to be a more relevant indicator of muscle function than is the thickness of the muscle. Because the observed correlations were moderate, these results warrant cautious interpretation. Nevertheless, integrating muscle morphology, muscle strength, and aerobic fitness assessments may contribute to a more comprehensive clinical evaluation. Taken together, these findings support a potential complementary role for chest CT in the early identification and monitoring of muscle alterations in patients with CF.
